# Surface Response Based Modeling of Liposome Characteristics in a Periodic Disturbance Mixer

**DOI:** 10.3390/mi11030235

**Published:** 2020-02-25

**Authors:** Rubén R. López, Ixchel Ocampo, Luz-María Sánchez, Anas Alazzam, Karl-F. Bergeron, Sergio Camacho-León, Catherine Mounier, Ion Stiharu, Vahé Nerguizian

**Affiliations:** 1 Department of Electrical Engineering, École de technologie supérieure, 1100 Notre Dame-West, Montreal, QC H3C 1K3, Canada; ruben.lopez-salazar.1@ens.etsmtl.ca (R.R.L.); anas.alazzam@ku.ac.ae (A.A.); vahe.nerguizian@ens.etsmtl.ca (V.N.); 2 School of Engineering and Sciences, Tecnológico de Monterrey, Av. Eugenio Garza Sada 2501 Sur, Monterrey 64849, N.L., Mexico; A00800468@itesm.mx (I.O.); sergio.camacho@tec.mx (S.C.-L.); 3 Department of Engineering, Universidad Autónoma de Querétaro Cerro de las Campanas s/n, Santiago de Querétaro 76010, Qro., Mexico; luzmsr@ieee.org; 4 System on Chip Center, Department of Mechanical Engineering, Khalifa University, Abu Dhabi 127788, UAE; 5 Department of Biological Sciences, Université du Québec à Montréal, 141 Président-Kennedy, Montreal, QC H2X 1Y4, Canada; bergeron.karl-frederik@uqam.ca (K.-F.B.); mounier.catherine@uqam.ca (C.M.); 6 Department of Mechanical and Industrial Engineering, Concordia University, 1455 de Maisonneuve Blvd. West, Montreal, QC H3G 1M8, Canada

**Keywords:** micromixers, liposomes, nanoparticles, microfluidics, continuous-flow synthesis

## Abstract

Liposomes nanoparticles (LNPs) are vesicles that encapsulate drugs, genes, and imaging labels for advanced delivery applications. Control and tuning liposome physicochemical characteristics such as size, size distribution, and zeta potential are crucial for their functionality. Liposome production using micromixers has shown better control over liposome characteristics compared with classical approaches. In this work, we used our own designed and fabricated Periodic Disturbance Micromixer (PDM). We used Design of Experiments (DoE) and Response Surface Methodology (RSM) to statistically model the relationship between the Total Flow Rate (TFR) and Flow Rate Ratio (FRR) and the resulting liposomes physicochemical characteristics. TFR and FRR effectively control liposome size in the range from 52 nm to 200 nm. In contrast, no significant effect was observed for the TFR on the liposomes Polydispersity Index (PDI); conversely, FRR around 2.6 was found to be a threshold between highly monodisperse and low polydispersed populations. Moreover, it was shown that the zeta potential is independent of TFR and FRR. The developed model presented on the paper enables to pre-establish the experimental conditions under which LNPs would likely be produced within a specified size range. Hence, the model utility was demonstrated by showing that LNPs were produced under such conditions.

## 1. Introduction

Liposomes are sphere-shaped vesicles with a range size from 20 nm to 2 µm. Liposomes are made of lipids that have an amphiphilic nature; this property allows them to self-assemble when an organic solvent, where initially they are dissolved in, is replaced by an aqueous solvent [1]. Liposomes are used for multiple biomedical applications such as drug delivery, medical imaging, gene delivery, and analysis [2]. 

The physicochemical properties of liposomes such as size, size distribution, and zeta potential determine how these vesicles interact with living entities. Inside the human body, liposome size influences to a high degree where liposomes will accumulate with a well-defined size range found to target specific organs [3], mostly the ones with the size below 150 nm. Accordingly, liposome size distribution is an essential factor as monodispersed liposome populations of the appropriate size will foster selective accumulation in higher percentages as opposed to those with highly polydispersed ones. Likewise, zeta potential is related to the net electric charge of the particle and the bulk fluid where particles are dissolved. Zeta potential is also known as electrokinetic potential. It determines the interaction between cells and particles, such as liposomes. These effects have been demonstrated in transfection applications [4,5]. Similarly, zeta potential values have shown to regulate the clearance rate in drug delivery applications where values range from -55 mV to 62 mV. A positive electrokinetic potential has shown to increase circulation time in *in vivo* models [6]. For these reasons, controlling final liposome characteristics is crucial for cargo delivery purposes.

The liposomes’ production method influences their final characteristics. Conventional liposome production methods such as thin-film hydration [7], ethanol injection [8], and reverse evaporation [9] usually lead to polydisperse liposomes, which require further homogenization steps such as sonication, extrusion, or solution handshaking [10]. These methods are complicated to scale-up and suffer from low batch to batch reproducibility. Micromixers offer an alternative method of liposome production, improving control over liposome properties. Typically, micromixers possess channels that are tens to hundreds of µm wide, where substances are mixed. At this scale, laminar flow commonly occurs, providing fluids with stable and uniform mixing interfaces, with mixing times in the millisecond range [11,12,13]. This mixing speed is essential for reactions where unstable intermediate states are produced as in the case of liposomes. Liposomes first form intermediate disk-shaped aggregates before self-assembly into spherical-shaped vesicles [14]. Furthermore, micromixers can operate in continuous flow conditions, increasing reproducibility. They can be easily parallelized in order to scale up production, resulting in process intensification as well as reducing waste and cost [15]. 

Micromixers based only on molecular diffusion or chaotic advection as mixing strategies have been extensively studied for liposome production [16,17]. However, these methods have shown limitations such as low final lipid concentration [16,18] and relatively poor device reliability [19]. Alternatives to such types of devices are Dean forces-based micromixers, which use curvilinear paths to induce centripetal forces, thus speeding up the mixing process [20,21,22]. Multiple micromixer designs using Dean forces have been previously proposed [19,23,24,25,26,27], showing promising results for lipid-based nanoparticles both in terms of increased yield (by reducing the Flow Rate Ratio (FRR) required to produce nanosized particles) as well as ease of fabrication of the microfluidic devices.

Furthermore, modeling and optimization of the produced liposome characteristics using advanced statistical tools have proved to be useful for finding the suitable flow conditions under which LNPs can be synthesized in micromixers such as the Staggered Herringbone Micromixer (SHM) for drug delivery systems [28,29] and the Micro Hydrodynamic Focusing Mixer (MHF) for gene delivery applications [30]. This statistical characterization is necessary, given the complicated relationship between the factors controlling liposomes’ properties. These properties vary from one micromixer to another, depending on their mixing principles, as well as other factors encountered at a molecular level [31]. To the best of our knowledge, no mathematical model connecting these variables has been established yet.

By contrast, such statistical methods have not been used in Dean forces-based micromixers other than in a study to assess the most influential variables among FRR, temperature, Total Flow Rate (TFR), lipid mixture, concentration, and micromixer geometry [32]. An advanced statistical study, as well as the use of these data for modeling, will provide useful information for controlling and modulating key liposome characteristics for biomedical applications. 

In this work, we used a Dean forces-based micromixer with a novel design using a curvilinear mixing channel to induce an alternatively changing force vector, called a Periodic Disturbance Mixer (PDM). We used Design of Experiments (DoE) and Response Surface Methodology (RSM) to model and optimize liposome size, size distribution, and zeta potential (for a complete list of abbreviations and symbols please refer to Supplementary Materials Tables S1 and S2). This methodology enables us to rapidly screen, optimize, and predict final liposome characteristics that otherwise would be time-consuming, e.g., study one factor at the time. We demonstrated that our model was able to predict an experimental region where size-controlled liposomes ranging from 52 nm to 200 nm were produced. Finally, through numerical modeling, our work offers a glance at the relationship between the mixing performance of the proposed device and the properties of liposomes.

## 2. Materials and Methods 

### 2.1. Micromixer Device Fabrication and Design

**Figure 1:**
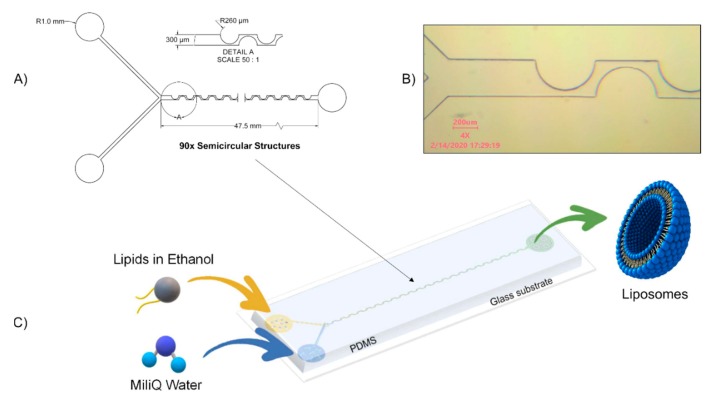
Periodic Disturbance Micromixer. (**A**) Microchannel dimensions. (**B**) Microscope image of the Periodic Disturbance Micromixer (PDM) device. (**C**) Schematic representation of the liposome formation process, lipids diluted in ethanol are injected in one inlet, while MiliQ water is injected in the second inlet. After passing through the mixing channel, liposomes are collected at the outlet.

The PDM micromixer consists of two inlets in one extremity that convey fluid from a Y-shape into a mixing channel with 90 semicircular structures and an outlet at the other extremity, as shown in Figure 1A. The semicircular structures center of curvature changes in such a way that the centripetal forces vector changes by 180° within the plane of the flow main advection direction. Their radius was chosen to achieve 90% mixing efficiency in the order of hundreds to tens of milliseconds according to numerical models developed and run in COMSOL Multiphysics 5.5 software as further detailed in Section 2.5 and Section 3.7. The microchannel height was designed to be as large as possible to increase the potential liposome production yield while considering the aspect ratio microfabrication constraint, which in practice is typically up to 1:10. The final micromixer dimensions are as follows: the mixing channel height, as well as width, is 300 µm; the semicircles along the structure have a radius of 260 µm and, consequently, the narrowest mixing channel portion is 40 µm. The total micromixing channel length is 47.5 mm, as shown in Figure 1A. The Y-shaped inlet channels and the first two semicircular structures are shown in Figure 1B. Liquids flow from left to right as shown in Figure 1C.

The microfluidic device was fabricated using a standard soft-lithography process. The microfluidic device is made of Polydimethylsiloxane (PDMS) (Ellsworth Adhesives Canada, Stoney Creek, ON, Canada) plasma bonded (Glow Research, Tempe, AZ, USA) to a 75 × 25 mm microscope slide (Globe Scientific Inc., Mahwah, NJ, USA). Tygon^®^ Tubing (Cole-Parmer, Montreal, QC, Canada) and stainless-steel fitters were used to connect the micromixer to syringes.

### 2.2. Lipid and Liposome Preparation

1,2-dimyristoyl-sn-glycero-3-phosphocholine (DMPC), cholesterol, and dicetyl phosphate (DHP) (Sigma-Aldrich, Oakville, ON, Canada) were mixed in a molar ratio 5:4:1. Reagents were first diluted in chloroform. Then the solvent was removed under an atmosphere free of oxygen using a nitrogen stream to avoid lipid degradation and to accelerate the process, as presented in previous works in the field [16]. The lipid mixture was stored under vacuum conditions for 24 h to remove solvent remnants. The mixture was then re-dissolved in ethanol to a final concentration of 5 mM. Then the lipid mixture was loaded into a syringe (Norm-Ject 10mL), while another syringe was loaded with ultrapure water filtered through the Mili-Q^®^ Direct water purification system (Millipore Sigma, Oakville, ON, Canada), which removes particles, salts, and bacteria. All previously described liquids were filtered using 0.22 µm filters. The syringes were connected separately to each of the inlets in the previously described microfluidic device (Figure 1C) through 0.22 µm filters to avoid bubble generation and debris. The flows were controlled using two syringe pumps Harvard Apparatus 11 plus 70-2212 (Harvard Apparatus Canada, Montreal, QC, Canada). Liposomes were collected at the outlet for a total volume of 4 mL. The process temperature was set to 70 °C using a hot plate on which the micromixer was placed, this value considered the lipids transition temperature and, at the same time, it was set below ethanol’s boiling point, which is 78 °C. Another reason to choose this temperature was that previous studies have shown that increasing temperature reduces liposome size [33]. Finally, the solution containing the liposomes was collected on the hotplate, then it was cooled down for 15 min at room temperature to enable liposomes to anneal. Samples were stored at 4 °C to avoid degradation and to increase shelf life.

TFR is defined as the sum of flows of the two inlets in the device, while FRR is defined as the MiliQ water to ethanol flow rate ratio. The flows in the microfluidic device were calculated as follows.

(1) $$Q_{\mathrm{as}}=\;\frac{\mathrm{FRR}\cdot \mathrm{TFR}}{\left(1\;+\;\mathrm{FRR}\right)}$$

(2) $$Q_{\mathrm{os}}\;=\;\frac{\mathrm{TFR}}{\left(1\;+\;\mathrm{FRR}\right)}$$

where Q_as_ is the aqueous solvent flow (MiliQ Water), Q_os_ is the organic solvent (ethanol plus diluted lipids), TFR is the total flow rate, and FRR is the flow rate ratio between the Q_as_ and Q_os_. More details about how Equations (1) and (2) were derived can be found in Appendix A.

### 2.3. Liposome Characterization

Liposome size and size distribution were measured using Dynamic Light Scattering. This technique traces Brownian motion and relates this motion to particle size. The equipment used was the Zetasizer Nano S90 (Malvern, Worcestershire, UK). The equipment utilizes a He/Ne laser with emission at 632.8 nm, a power of 4 mW, and a 90° scattering detector. Measurements were performed at a stable temperature of 25 °C. Five hundred microliters of each sample was placed in a low-volume disposable cuvette. The average hydrodynamic diameter (Z-Average) and Polydispersity Index (PDI) of at least three independent measurements were recorded per sample. Liposomes collected from the outlet were further diluted for a final lipid concentration of 0.04 mg/mL. This concentration proved to yield high-quality measurement results. Liposome zeta potential was measured by placing each sample in a disposable cuvette (1850 µL). The measurements were performed using the ZetaPlus (Brookhaven Instrument Corp., Holtsville, NY, USA) at a stable temperature of 25 °C and a pH = 7.00. The measurement results were calculated based on the electrophoretic mobility of suspended particles under an electrical changing field. Zeta potential average is a result of 2 stable cycles and ten measurement repetitions.

### 2.4. Design of Experiments

Liposome final characteristics such as size, size distribution, and zeta potential relationship with flow conditions were modeled using RSM and DoE. RSM enables the evaluation of multiple factors or variables and their interactions influence on one or more responses, using regression analysis. This methodology allows us to predict and optimize process responses [34]. The use of DoE as opposed to One Factor At the Time (OFAT) allows interpreting interactions of two variables at the same time, as well as reducing the number of required experiments to model the liposome production process and time. In this work, TFR and FRR are considered factors independent process variables, while Z-Average (liposome diameter), PDI (size distribution), and zeta potential are considered responses.

The region of interest for the variables was delimited, considering the micromixer operational integrity and conditions under which a stable flow regime could be achieved. Table 1 shows the initial low and high values for each factor. 

A two-variable Central Composite Circumscribed Rotatable (CCCR) Design was created using the software Minitab^®^ 19, considering the values in Table 1. This experimental design predicted that the variance is only dependent on the distance from the center point. This design is used for quadratic models. 

**Figure 2:**
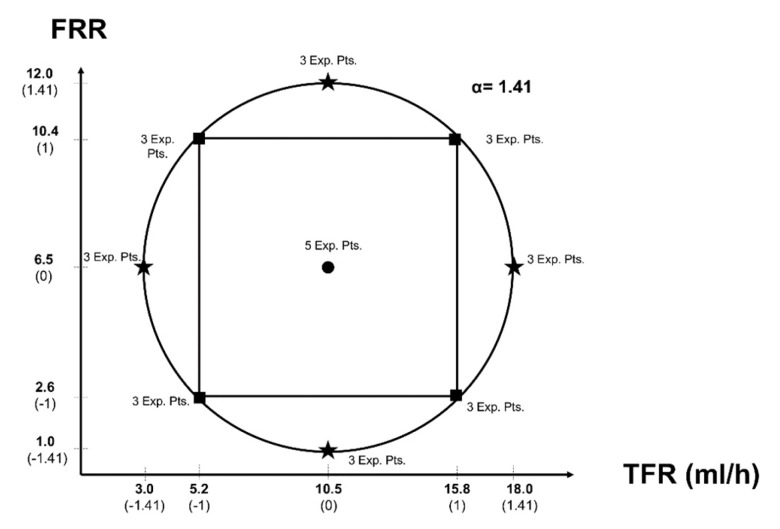
Central Composite Circumscribed Rotatable (CCCR) Design. All experimental points are at the same distance from the center. The experimental design has four axial points and four cubic points, each one with three repetitions and one central point with five repetitions. α = 1.41. Coded values are in parentheses.

The optimized experimental design resulted in 29 experimental points, 4 axial points with 3 repetitions each that are the extreme values above and below the low and high settings, 4 cube points with 3 repetitions each that are the initial low and high values, and one center point with 5 repetitions that are in the middle between the cube points. The value α, which represents the distance between the center point and an axial point in coded units. The value was calculated as follows:

(3) $$\alpha \;=\;{\left[2^{k}\right]}^{\frac{1}{4}}$$

where *k* is the number of factors, in this case, 2 for a final α = 1.41. The order of experimental runs was randomized. The continuous value for each experimental point was calculated by converting the coded value scale to the continuous value. Due to pumps resolution, values were rounded to 1 decimal position. Figure 2 shows the graphical representation of the experimental space and points for the two factors TFR and FRR.

Finally, a surface response model of second order with interactions was fitted for Z-average and PDI. Later, the model was assessed using Analysis of Variance (ANOVA) to identify the goodness of fit of R^2^, R^2^ (predicted), and model significance as well as each of the terms predicting liposome size and PDI (*p* = 0.05). The best model, among them, was chosen considering the statistics results. A post hoc Tukey test *p* = 0.05 was used to evaluate PDI and zeta potential groups. A paired t-test was used to assess liposome stability statistically. All previously described tests were performed using the software Minitab^®^ 19.

### 2.5. Mixing Efficiency Using Numerical Modeling

The micromixing phenomenon was numerically modeled using Navier-Stokes equations coupled with the convection–diffusion equation, considering a single-phase flow using the software COMSOL Multiphysics 5.5. The next set of equations was solved until a steady state was reached.

(4) $$\rho \left(u\cdot \nabla \right)u\;=\;\nabla \cdot \left[-\mathrm{pI}\;+\;\mu \left(\nabla u+{\left(\nabla u\right)}^{T}\right)\right]\;+\;F$$

(5) ρ∇·(u) = 0

Where ρ is the fluid density, u  is the flow velocity, p is the pressure, μ is the dynamic viscosity, and F represents outer forces. The boundary conditions for the walls were set to no-slip condition. The velocity field that resulted from previous equations was used to solve the following set of equations, including the convection–diffusion equation.

(6) ∇·(−D∇c) + u·∇c = R

(7) N = −D∇c + uc

where c is the diluted species concentration, D is the mutual diffusion coefficient between water and ethanol, R is the net volumetric source for the species, and N is the molar flux. 

In previous equations, D, ρ, and µ are in function of the solvent concentration c in the aqueous media. This approach allowed it to use a single-phase liquid with variable fluid characteristics to model a binary mixture. The equations approximating the mutual diffusion coefficient are described in the model by a 5th order polynomial fitting experimental data from the binary mixture ethanol–water [35,36].

Mixing Efficiency (ME) inside the channel was calculated by first dividing the given cross-sections normal to the main flow direction into a grid of 50 × 50 elements. Then, the Danckwert´s intensity of segregation concept was applied to the concentration of these cross-section elements [37]. ME was calculated using Equation (8).

(8) $$\mathrm{ME}\;=\left[1-\sqrt{\frac{\sigma^{2}}{\sigma_{0}{}^{2}}}\right]\times \;100\%$$

where $\sigma_{0}{}^{2}$ is the variance of concentration at the beginning of the mixing channel, where liquids are completely unmixed and $\sigma^{2}$ is the variance of the concentration at a given cross-section.

## 3. Results and Discussion

The liposome characterization results from the CCDR experimental design were used to create through response surface methodology, a model capable of predicting Z-average, PDI, and zeta potential. Finally, model predictions were compared with an experimental validation run. These results were discussed and related to mixing efficiencies in the PDM.

### 3.1. Liposome Final Characteristics DoE results

We investigated the influence of FRR and TFR using the experimental design shown in Table 2. The coded values are in parenthesis. The resulting responses: i.e., liposome size (Z-average), PDI, and Zeta Potential, are given on the right side of the same table. 

Liposome size ranged from 52 nm to 200 nm for the tested experimental conditions. PDI oscillated between 0.060 (highly monodisperse populations) to a maximum of 0.270 (low polydispersed populations). Lastly, zeta potential varied in a range from −37.7 mV to −24.6 mV.

Liposomes produced under the same experimental conditions showed a maximum standard deviation of 11.48 nm in size and a minimum of 0.43 nm. These variations might have been caused by batch to batch lipid preparation variations and unpredicted bubbles generated inside mixing channels that can disturb the stability of the flow’s interface.

### 3.2. Liposome Size (Z-Average) Modeling

First, a full quadratic surface response model was fitted using FRR and TFR as independent factors and Z-average (size) as a response. A one-way ANOVA analysis was performed for each term in the model. Then the model was reduced, considering the level of significance of each term. All terms with a *p* > 0.05 were ignored. A new surface response model was run to increase the prediction precision or R^2^-predicted. Equation (9) shows the final surface response model. Note that TFR^2^ and TFR·FRR were removed because they yielded a *p* > 0.05.

(9) $$Z_{\mathrm{average}}\;=\;236.3\;-\;26.95\;\mathrm{FRR}\;-4.437\;\mathrm{TFR}\;+\;1.573{\left(\mathrm{FRR}\right)}^{2}$$

**Figure 3:**
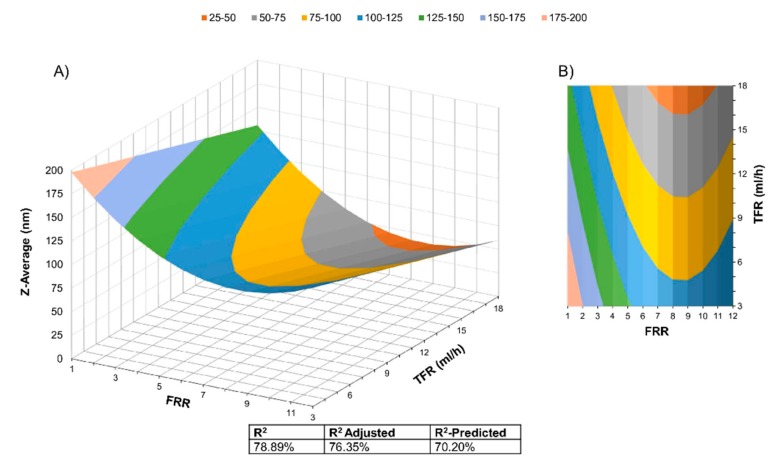
Surface Response Model derived from the Central Composite Circumscribed Rotatable Design Data. The model level of significance is *p* < 0.05. (**A**) 3-dimensional, (**B**) Contours representation.

Figure 3 shows the surface response model derived from the data, as well as the R^2^, R^2^-adjusted, and R^2^-predicted. The linear factors TFR and FRR reduce liposome size; on the other hand, FRR^2^ avoids further liposome size reduction for a value above 8.56.

The factors FRR and TFR showed a considerable influence over final liposome size, this in accordance with previous works in the field for other types of mixers such as the Micro Hydrodynamic Focused (MHF) micromixer [38], the Staggered Herringbone Micromixer [17], and Dean Forces based micromixers [19,24]. Compared to other statistical models for SHM and MHF geometries [29,30], the R^2^ values are similar. Our model did not show curvature for TFR, indicating a simple linear interaction related to this variable and liposomes size. Considering that drug delivery systems require liposomes with a size range from 45 nm (DauXome^®^) to 180 nm (Myocet^®^) [28], we used the model to identify the flow conditions that could lead to this size range with an emphasis in finding the minimum realizable liposome size. 

We used the model and optimized the factor values without restrictions within the experimental range to minimize the function, which, in this case, is liposome size (Z-average). The minimum average size predicted was 41.07 nm, with a 95% confidence range between 25.10 nm and 57.03 nm. The factors levels are FRR = 8.56 and TFR = 18 mL/h.

### 3.3. Polydispersity Index Modeling

The PDI range in the experimental results is from 0.06 to 0.27, or from highly monodisperse populations to low polydispersed populations. These values are comparable with MHF and SHM micromixers for liposome production.

We proceeded using a similar approach to analyze the influence of the two factors, FRR and TFR, over the response PDI. In this case, we also eliminated terms that are not statistically significant (*p* > 0.05), as in the previous model. The quadratic term TFR^2^ and the interaction term TFR·FRR were eliminated. Equation (10) shows the final model with four terms.

(10) $$\mathrm{PDI}\;=\;0.0663\;+\;0.03181\;\mathrm{FRR}\;+\;0.00319\;\mathrm{TFR}\;-0.001905{\;(\mathrm{FRR})}^{2}$$

**Figure 4:**
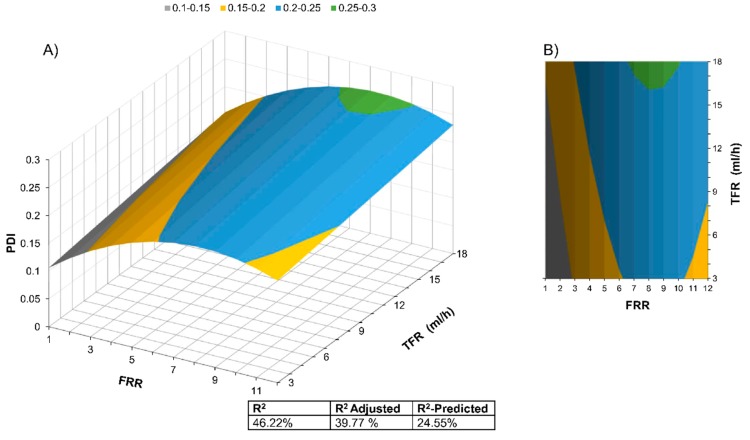
PDI Surface Response Model as well as the R^2^, R^2^-adjusted, and R^2^-predicted. The model has a *p* < 0.05. (**A**) 3-dimensional, (**B**) Contours representation.

The surface response model for PDI is shown in Figure 4. Equation (10) shows that the value of the PDI is proportional to the FRR^2^ and TFR. Most of the model results for PDI are ranging between 0.1 and 0.3 for the experimental range.

To better understand the reasons behind R^2^ results, we performed a Tukey pairwise comparison to group the significantly different PDI means (95% confidence level), as shown in Table 3.

The comparison detected three entirely statistically different conditions among the nine tested in this work for the PDI, with a mean of 0.07, 0.18, and 0.24. It could be inferred from the experimental conditions that a low FRR value results in highly monodisperse populations; however, above FRR = 2.6, the PDI values are mostly the same and only when FRR = 12.0, another PDI population appears. This behavior might indicate that the PDM has well-delimited operation regimes.

### 3.4. Zeta Potential Relationship with TFR and FRR

**Figure 5:**
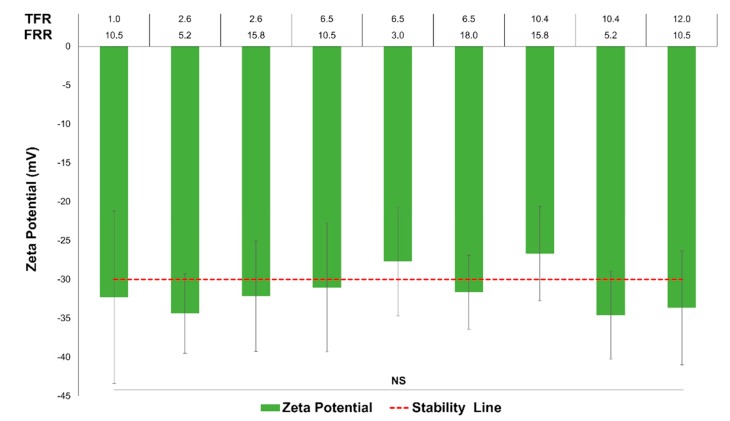
Zeta potential measurements result for the nine different conditions. The *x*-axis values correspond to TFR on the top and FRR on the bottom. The red line marks the stability line for particle dispersions (+/− 30 mV). The error bars show 1.96σ, indicating the limits of 95% confidence.

Finally, the zeta potential values range was limited from −38.8 to −23.1 mV on average. The means of each condition are not statistically significantly different from each other, according to grouping information using the Tukey method and 95% confidence. Figure 5 shows the zeta potential for the nine different conditions. This factor showed itself to be independent of TFR and FRR; thus, the model was not significant.

### 3.5. Statistical Model for Controlling Liposome Characteristics

The statistical information, together with the models, indicate that liposome size could be tuned by modifying TFR and FRR. On the other hand, PDI has three different groups, which might be related to the micromixer operation regime. Finally, neither the TFR nor FRR has any influence over zeta potential results using a PDM. Results from other types of micromixers indicate that only composition plays a role in liposome zeta potential [5]. Table 4 shows a summary of the statistical models.

### 3.6. Model Prediction vs. Experimental Validation Run

**Figure 6:**
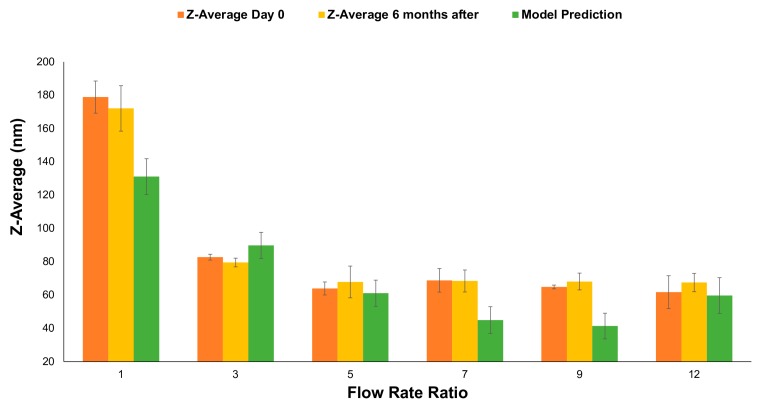
Z-average (nm) vs. FRR at a constant TFR = 18 mL/h. In orange, the Z-average immediately after production, in yellow six months after, and in green the model prediction. *n* = 3. Error bars indicate +/− 1 standard deviation (SD) for samples and SE fit for the model prediction.

The flow conditions which the model predicted would lead to the minimum liposome size were validated, as were the conditions in the vicinity of the prediction. The model predicted a minimum size at approximately FRR = 9 and TFR = 18 mL/h. The predicted size was 41 nm. Figure 6 shows the size immediately after production, after six months, and the predicted size using the model presented previously. The model predicted, with reasonable accuracy, the size for an FRR of 3, 5, and 12. The size for FRR of 1, 7, and 9 was out of the Standard Error (SE) fit value. Liposome size reduction reaches a plateau before reaching high FRR values. These results may represent the limit for the presented conditions and the PDM. This size plateauing at high FRR is found in other types of micromixers such as the Vertical Flow Focusing Device (VFF) [39] and recently published 3D printed devices for liposome production [40]. The asymptotic behavior of the liposome size is challenging to predict using a quadratic model.

Moreover, there was not a statistical difference in liposome size six months after production. This result indicates the high stability of the liposome nanoparticles produced, which suggests a suitable shelf life of such a product.

**Figure 7:**
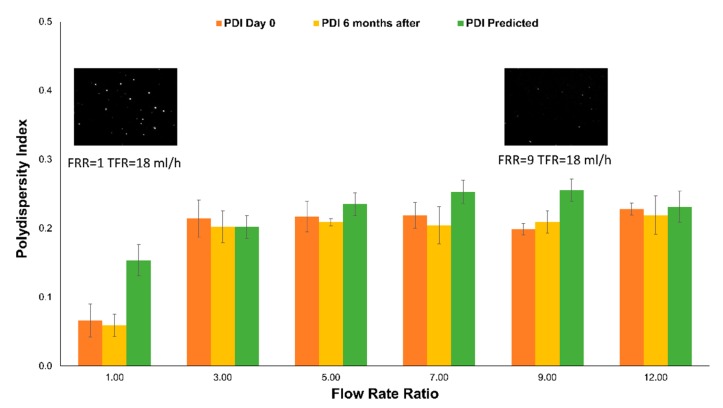
Liposomes PDI measurements immediately after production (orange), six months after (yellow), and the model prediction (green) at a constant TFR = 18 mL/h. *n* = 3, error bars indicate +/− 1 SD for samples and SE fit for the model prediction. Images are taken from videos using NTA, as shown in supporting material Videos S1 and S2.

Finally, the PDI for measurements performed immediately after liposome production, six months after, and the model predictions were compared, as shown in Figure 7. The same figure shows the snapshots of videos taken using Nanoparticle Tracking Analysis (NTA) of two samples produced at FRR = 1 and FRR = 9 (Videos S1 and S2). The comparison between DLS and NTA results are shown in Table S3. In this case, even if the model is not a good fit (R^2^-predicted = 24.55), it predicted values that are close to the measured values due to the low variation of PDI and because of the change induced by FRR is minimal. It could be considered that PDI is independent of the experimental range for values above FRR = 3, indicating an intrinsic process property. Number-wise, PDI values do not change significantly. However, this variation has an impact on liposome population classification between monodispersed and low polydispersed populations.

### 3.7. Relationship Between the Mixing Process and Liposome Properties

To investigate further Z-average and PDI results, the mixing efficiencies inside the microchannel were evaluated using the numerical model detailed in Section 2.5. The experimental mixing performance of the device in function of FRR is shown in Videos S3–S8. Table S4 shows the flow conditions for each video. Liposomes are formed when lipids initially diluted in ethanol agglomerate first in intermediate disk-shaped structures due to the polarity change caused by the mix of ethanol and water. As the polarity continues to increase, as a consequence of a lower ethanol concentration in the binary mixture, these disk-shaped agglomerates are forced to close into spheres to avoid lipids tails exposure to a high polarity value media [41]. How fast the transition occurs from agglomeration to self-assembly highly determines liposome size [42]. The mixing speed and uniformity modulate this transition from low polarity to high polarity.

**Figure 8:**
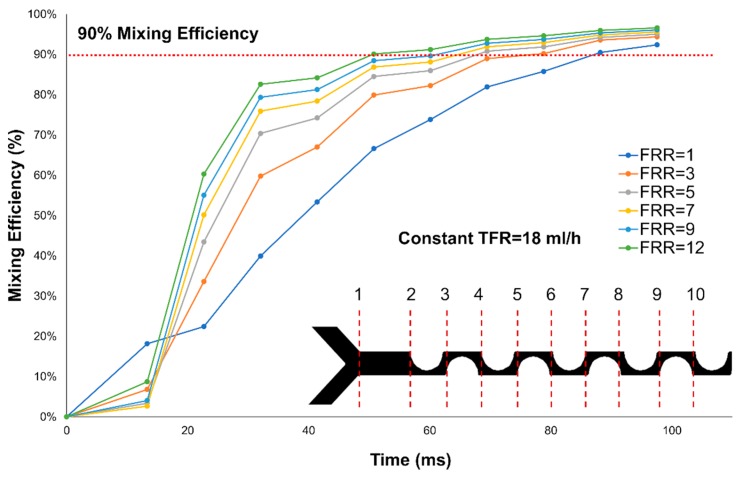
Mixing efficiency at different FRRs. Each data point corresponds to a cross-section for a total of 10 data points from 1–10.

In the presented validation experiments, FRR modulated the speed at which ethanol and water mixed, as illustrated in Figure 8. The separation between where each condition crosses 90% of mixing efficiency is reduced at each step. This feature closely relates to liposome size decrease as shown in Figure 6. Moreover, the slope of the curve for FRR = 1 is very different compared to all the other conditions. The latter indicates that PDI might be modulated by the speed at which mixing efficiency increases.

**Figure 9:**
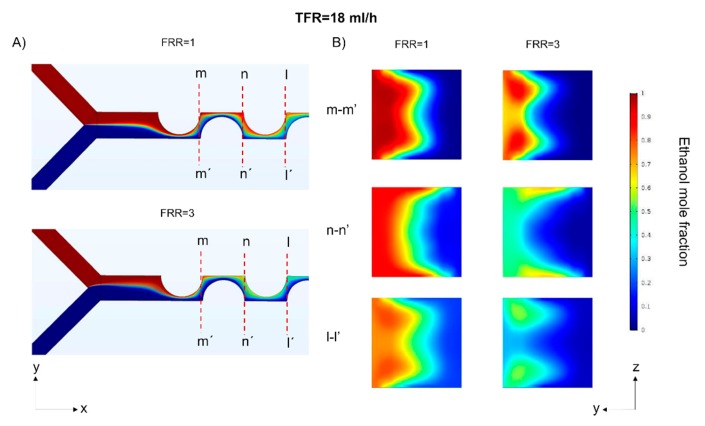
Numerical simulations comparing the concentration profiles at FRR = 1 and FRR = 3 at a constant TFR = 18 mL/h. (**A**) Upper view of the mixing channels and position of the cross-sections. (**B**) Cross-sections at different FRRs.

The relationship between concentration profiles uniformity and PDI has been investigated for VFF mixers [39], where the aspect ratio controls the concentration profile uniformity. By contrast, in the PDM, FRR modulates the profile uniformity. PDI results indicate that a threshold value separating monodisperse populations and low polydisperse populations exist at FRR ≥ 3. These results could be related to a more uniform mixing profile at FRR = 1 compared with other FRR values. FRR defines the width of the diluted species at the beginning of the channel. Higher FRRs values mean a narrower ethanol interface, which allows a faster dispersion perpendicular to the main advection direction resulting in smaller liposomes. Although higher FRRs results in non-uniform concentration profiles that might result in more polydisperse populations. This difference in the concentration profile is illustrated in Figure 9 in different cross-sections for FRR = 1 and FRR = 3. 

## 4. Conclusions

In this work, we used DoE and RSM to model, control, and optimize liposome properties in a Periodic Disturbance Mixer. FRR and TFR control liposome Z-average (size), as the model predicted. On the other hand, PDI showed three statistically distinct mean values, indicating likely operating regime ranges. One of these PDIs is remarkably different from the other two at low FRR. This behavior could suggest that centripetal forces start to produce Dean vortices only after specific flow conditions are reached in our proposed micromixer. Lastly, zeta potential values under different experimental conditions are not statistically different from each other, indicating that FRR and TFR do not influence this variable. These results also suggest that the liposomes’ zeta potential is controlled by another factor not studied in this work. 

The size range of liposomes produced by our proposed device is comparable to currently available liposome formulations in the market.

Additionally, through numerical simulations, we investigated the relationship between the mixing conditions in the PDM and liposome properties such as size and PDI. Mixing time was related to liposome size, while the uniformity of the mixing profile was related to PDI.

Further studies are needed to understand why there are different flow regimes affecting size and PDI. Moreover, other properties influencing liposomes’ self-assembly at a molecular level should be addressed.

## Figures and Tables

**Table 1:** Initial low and high experimental levels per variable.

Name	Type	Low (Coded Value)	High (Coded Value)
**FRR**	Continuous	1.0 (−1)	12.0 (1)
**TFR**	Continuous	3.0 mL/h (−1)	18.0 mL/h (1)

**Table 2:** CCCR design results for the 29 runs. In parenthesis, the coded values are given. The factors are Flow Rate Ratio (FRR) and Total Flow Rate (TFR). The coded values are in parenthesis, as shown in Figure 2. The responses are the size (Z-average), Polydispersity Index (PDI), and zeta potential.

**Run Order**	**Factors**		**Responses**		
	**FRR**	**TFR (mL/h)**	**Z-Average (nm)**	**PDI**	**Zeta Potential (mV)**
**1**	6.5 (0)	3.0 (−1.41)	133.50	0.185	−31.6
**2**	1.0 (−1.41)	10.5 (0)	190.70	0.060	−38.8
**3**	10.4 (1)	15.8 (1)	67.52	0.202	−23.1
**4**	6.5 (0)	18.0 (1.41)	66.63	0.185	−29.8
**5**	12.0 (1.41)	10.5 (0)	75.09	0.232	−37.9
**6**	10.4 (1)	5.2 (−1)	133.5	0.174	−32.1
**7**	2.6 (−1)	5.2 (−1)	119.40	0.223	−35.2
**8**	2.6 (−1)	15.8 (1)	86.48	0.217	−29.9
**9**	6.5 (0)	10.5 (0)	81.81	0.207	−32.3
**10**	6.5 (0)	3.0 (−1.41)	120.70	0.179	−24.6
**11**	1.0 (−1.41)	10.5 (0)	197.00	0.072	−28.5
**12**	10.4 (1)	15.8 (1)	62.10	0.270	−28.8
**13**	10.4 (1)	5.2 (−1)	120.20	0.170	−33.9
**14**	6.5 (0)	18.0 (1.41)	57.14	0.238	−33.8
**15**	12.0 (1.41)	10.5 (0)	74.14	0.245	−30.7
**16**	2.6 (−1)	5.2 (−1)	122.4	0.207	−31.5
**17**	2.6 (−1)	15.8 (1)	88.74	0.221	−36.3
**18**	6.5 (0)	10.5 (0)	72.23	0.230	−37.7
**19**	6.5 (0)	10.5 (0)	73.81	0.235	−27.6
**20**	6.5 (0)	3.0 (−1.41)	116.00	0.189	−26.9
**21**	1.0 (−1.41)	10.5 (0)	199.70	0.064	−29.6
**22**	10.4 (1)	15.8 (1)	52.71	0.228	−28.2
**23**	10.4 (1)	5.2 (−1)	110.4.	0.184	−37.7
**24**	6.5 (0)	18.0 (1.41)	52.14	0.265	−34.4
**25**	12.0 (1.41)	10.5 (0)	73.80	0.247	32.4
**26**	2.6 (−1)	5.2 (−1)	131.60	0.206	−36.5
**27**	2.6 (−1)	15.8 (1)	90.27	0.241	−30.2
**28**	6.5 (0)	10.5 (0)	77.18	0.223	−27.6
**29**	6.5 (0)	10.5 (0)	77.24	0.262	−30.1

**Table 3:** Grouping information using the Tukey method at a 95% confidence level.

Condition	N	Mean	Grouping		
9 (FRR = 12.0 TFR = 10.5)	3	0.24	A		
6 (FRR = 6.5 TFR = 18.0)	3	0.24	A	B	
4 (FRR = 6.5 TFR = 10.5)	5	0.23	A	B	
3 (FRR = 2.6 TFR = 15.8)	3	0.22	A	B	
2 (FRR = 2.6 TFR = 5.2)	3	0.22	A	B	
7 (FRR = 10.4 TFR = 15.8)	3	0.21	A	B	
8 (FRR = 10.4 TFR = 5.2)	3	0.18		B	
5 (FRR = 6.5 TFR = 3.0)	3	0.18		B	
1 (FRR = 1.0 TFR = 10.5)	3	0.07			C

**Table 4:** Statistically significant coefficients for each model (*p* < 0.05) and model statistics summary.

Response	Significant Coefficients	R^2^	*p*-Value	*F*-Value
Size	FRR, FRR^2^, TFR	78.89%	1 × 10^−8^	31.14
PDI	FRR, FRR^2^, TFR	46.22%	0.001	7.16
Zeta Potential	None	NA	NA	NA

## Supplementary Materials

The following are available online at https://www.mdpi.com/2072-666X/11/3/235/s1, Supplementary Information. Table S1: List of abbreviations, Table S2: List of symbols, Table S3: DLS vs. NTA measurements, Table S4: Video names and flow conditions, Video S1: NTA FRR = 1 TFR = 18, Video S2: NTA FRR = 9 TFR = 18, Video S3: FRR = 1 TFR = 18, Video S4: FRR = 3 TFR = 18, Video S5: FRR=5 TFR = 18, Video S6: FRR = 7 TFR = 18, Video S7: FRR = 9 TFR = 18, and Video S8 FRR = 12 TFR = 18.

## Appendix A

**Flow Conditions Calculations**

The Total Flow Rate (TFR) is defined as the sum of the flows entering the microchannels. In this case, the aqueous solvent flow (MiliQ water) Q_as_ and the organic solvent flow (ethanol) Q_os_.

(A1) $${\mathrm{TFR}\;=\;Q}_{\mathrm{as}}{+\;Q}_{\mathrm{os}}$$

The Flow Rate Ratio (FRR) is defined as the ratio between the aqueous solvent and the organic solvent.

(A2) $$\mathrm{FRR}\;=\;\frac{Q_{as}}{Q_{os}}$$

Solving for Q_as_ and substituting in Equation (A1).

(A3) $$\mathrm{TFR}\;=\;\mathrm{FRR}\cdot Q_{os}{+\;Q}_{os}$$

Solving for Q_os_

(A4) $$Q_{os}\;=\;\frac{TFR}{1+FRR}$$

Finally, solving for Q_as_ in Equation (A4)

(A5) $$Q_{as}\;=\;\frac{FRR\cdot TFR}{\left(1+FRR\right)}$$

The flow in each of the inlets is defined in terms of FRR and TFR, as stated in Equations (1) and (2) in Section 2.2.
