# Supplementary material for: Surface Response Based Modeling of Liposome Characteristics in a Periodic Disturbance Mixer
Source: Micromachines (Basel). 2020 Feb 25;11(3):235. doi: 10.3390/mi11030235 (PMC7143066; doi:10.3390/mi11030235)
Supplement: Supplementary file 1 [file micromachines-11-00235-s001.zip › Supplementary Information.pdf]

Supplementary Information

Surface Response Based Modeling of Liposome Characteristics in a Periodic Disturbance Mixer

[1] Department of Electrical Engineering, École de technologie supérieure, 1100 Notre Dame-West, Montreal, Quebec, Canada; ruben.lopez-salazar.1@ens.etsmtl.ca, vahe.nerguizian@ens.etsmtl.ca
[2] School of Engineering and Sciences, Tecnológico de Monterrey, Av. Eugenio Garza Sada 2501 Sur, Monterrey, N.L., Mexico; A00800468@itesm.mx; sergio.camacho@tec.mx
[3] Department of Engineering, Universidad Autónoma de Querétaro Cerro de las Campanas s/n, Santiago de Querétaro, Qro., México; luzmsr@ieee.org
[4] Department of Mechanical Engineering, Khalifa University, Abu Dhabi, UAE; anas.alazzam@ku.ac.ae
[5] Department of Biological Sciences, Université du Québec à Montréal, 141 Président-Kennedy, Montreal, Quebec, Canada; bergeron.karl-frederik@uqam.ca; mounier.catherine@uqam.ca;
[6] Department of Mechanical and Industrial Engineering, Concordia University, 1515 Saint Catherine West, Montreal, Quebec, Canada; ion.stiharu@concordia.ca

* Correspondence author: ion.stiharu@concordia.ca; Tel.: +1-514-848-2424 Ext. 3152 (I.S.)

1. List of Abbreviations and Symbols

The next tables show the abbreviations and symbols used in the manuscript.

Table S1 List of abbreviations.

Abbreviation	Meaning
**ANOVA**	Analysis of Variance
**CCCR**	Central Composite Circumscribed Rotatable Design
**DHP**	Dicetyl phosphate
**DLS**	Dynamic Light Scattering
**DMPC**	1,2-dimyristoyl-sn-glycero-3-phosphocholine
**DoE**	Design of Experiments
**FRR**	Flow Rate Ratio
**LNP**	Liposome Nanoparticles
**MHF**	Micro Hydrodynamic Focusing Mixer (MHF)
**NTA**	Nanoparticle Tracking Analysis.
**OFAT**	One Factor At the Time
**PDI**	Polydispersity Index
**PDM**	Periodic Disturbance Mixer
**PDMS**	Polydimethylsiloxane
**SE**	Standard Error

Table S2 List of symbols.

Symbol	Meaning
$\boldsymbol{Q_{as}}$	Flow of aqueous solvent.
$\boldsymbol{Q_{os}}$	Flow of organic solvent
$\boldsymbol{\rho}$	Flow Density
**p**	Pressure
**u**	Flow Velocity
$\boldsymbol{\mu}$	Dynamic viscosity
**F**	Outer forces
**D**	Mutual diffusion coefficient
**c**	Concentration
$\boldsymbol{\sigma_0}$	Standard deviation at the beginning of the mixing channel
$\boldsymbol{\sigma}$	Standard deviation in the cross-section
**ME**	Mixing efficiency

2. Nanoparticle Tracking Analysis (NTA) Results

To compare with Dynamic Light Scattering (DLS) measurements and obtain a visual representation of the particles' Brownian motion, we used Nanoparticle Tracking Analysis (NTA) to characterize representative samples. This method gives a number-based particle size result. The difference in size is related to particle mobility. More mobility corresponds to a smaller sized particle. DLS and NTA measurements are shown in **Table S3** as well as the NTA videos reference.

Table S3 DLS vs. NTA measurements.

	**Flow Conditions**		**DLS**		**NTA**		
**Video Name**	**FRR**	**TFR (mL/h)**	**Z-Average (nm)**	**PDI**	**Mean (nm)**	**Mode (nm)**	**SD (nm)**
Video S1	1	18	184.7	0.045	189.8	130.0	68.5
Video S2	9	18	64.28	0.194	63.5	40.0	55.7

3. PDM Performance under Different FRR conditions

The next set of videos shows the PDM performance under different FRR conditions. Fluids flow from left to right. On the top channel, ethanol is injected while at the bottom, one water is injected. The next table shows the video reference as well as the flow conditions of such videos.

Table S4 Video names and flow conditions.

Video Name	FRR	TFR (mL/h)
Video S3	1	18
Video S4	3	18
Video S5	5	18
Video S6	7	18
Video S7	9	18
Video S8	12	18
